# Dairy farmer perceptions of antibiotic transport and usage in animal agriculture dataset

**DOI:** 10.1016/j.dib.2021.106785

**Published:** 2021-01-22

**Authors:** Christine B. Georgakakos, Betsy Hicks, M. Todd Walter

**Affiliations:** a Department of Biological and Environmental Engineering, Cornell University, 111 Wing Drive, Ithaca, NY 14850, United States; b Cornell Cooperative Extension, Ithaca, NY, United States

**Keywords:** Qualitative data, Interview, Contaminant loads, Emerging contaminant, Pharmaceutical

## Abstract

These data were from semi-structured interviews with dairy farmers. The content of the interviews focused on antibiotic transport and usage on dairy farms. Twenty-seven interviews were conducted in Central New York in 2019. Interviews were recorded and subsequently transcribed for qualitative thematic analysis. Qualitative coding analysis was preformed using ATLAS.ti and content filtered to ensure farmer anonymity. The dataset includes direct quotations from dairy farmers paired with farm and farmer characteristics. Quotations are subdivided thematically into the themes of disease prevention, antibiotic usage, non-antibiotic treatments, antibiotic transport, and environmental residue presence impacts, as structured in Georgakakos et al. [1]. Farm characteristics include management practice, farm size, and farm generation. Farm size was determined by number of lactating cows: small (0-50), medium-small (51-100), medium (101-500), medium-large (501-1000), and large (>1000). Farmer characteristics were farmer age categorized by birth year: Baby Boomer (1946-1964), Gen X (1965-1980), and Millennial (1981-1996). This dataset is particularly promising for longitudinal studies, incorporation of human behaviour into contaminant load models, or for recoding and analysis for themes other than those discussed by Georgakakos et al. [1].

**Specifications Table**

**Table:** 

Subject	Environmental Science: Pollution
Specific subject area	Farmer perceptions of antibiotic residue transport and usage on dairy farms in upstate New York
Type of data	Table
How data were acquired	Semi-structured interviews were recorded on site using a cell phone, and subsequently coded for themes using ATLAS.ti software Coding software: ATLAS.ti Software version: ATLAS.ti 8.4.5 for mac
Data format	Raw Filtered
Parameters for data collection	Parameters for inclusion in the dataset were: farms were located in central upstate New York, and farms produced a dairy product from dairy cows. All farms were in operation between January 2019 and July 2019.
Description of data collection	All interviews were conducted in person by the first author, recorded, and professionally transcribed by Cornell Institute of Survey and Economic Research. Interviews lasted between 21 and 87 min. Twenty seven interviews were conducted in total.
Data source location	Institution: Cornell University City/Town/Region: Ithaca, NY Country: USA
Data accessibility	http://dx.doi.org/10.17632/cf2wvytr5r.1
Related research article	C.B. Georgakakos, B.J. Hicks, M.T. Walter. Farmer perceptions of dairy farm antibiotic use and transport pathways as determinants of contaminant loads to the environment, *Journal of Environmental Management.* Volume 281,1 March 2021,11180. doi.org/10.1016/j.jenvman.2020.111880

**Value of the Data**  

• Qualitative raw data are infrequently published. Antibiotic residue transport and spread antimicrobial resistance are of increasing concern, with a lack of both quantitative and qualitative data publicly available.
• These data can benefit future researchers in guiding new research directions, extension educators/outreach personnel to focus educational programs, and policy initiatives aimed to reduce spread of antimicrobial resistance.
• These data are useful for inclusion of human behaviours and drivers in contaminant models tracing pharmaceutical loads into soil and water systems from agriculture as well as for researchers building upon themes that emerged in these interviews to direct future surveys and data collection. Additionally, interviews could be recoded for novel themes.
• This dataset is especially useful in the case of a repetitive, longitudinal study to assess changes in antibiotic transport and usage perceptions over time and across differing policy environments.

## 1 Data Description

**Dataset**: Full interview transcripts have not been published for to ensure confidentiality of included farmers. Direct quotations used for analysis in Georgakakos et al. [1], have been sorted by interview number and theme. Descriptive data on each interview has been tabulated and included. Any identifying information included in these quote pulls were removed from the dataset and replaced with ‘XXXX’. ‘I:’ indicates text from the interviewer, ‘FR:’ indicates text from a female respondent, and ‘MR:’ indicates text from a male respondent. Noncontinuous quotations are separated by ‘…’. The data included relate to antibiotic residue pathways through dair farm systems, and dairy farmer decisions that intentional or unintentionally modify environmental antibiotic loading.

**Supplementary Material: Table 1**: Interview Guide. This semi-structured interview guide was developed from the interview guide developed by Wemette et al. [2]. Nine of the 37 interview questions were taken from Wemette et al [2]. The questions that are consistent between Wemette et al, [2] and Georgakakos et al., [1] are predominantly background and introductory questions used to locate the type of operation each farm employs. Georgakakos et al. [1] focus heavily on antibiotic residue pathways into the environment, a direction not explored by Wemette et al. [2].

**Supplementary Material: Table 2**: Code book. This table includes all associated codes, search words, and instances of occurrence throughout all interviews. All code words included in this table are not analyzed by Georgkakos et al. [1] and all associated quotations do not appear in the dataset.

## 2 Experimental Design, Materials and Methods

We conducted semi-structured interviews on dairy farms in central New York state. For inclusion in this dataset, farms were required to be located in central New York State, and produce a dairy product from cows. Farms were not required to rely entirely upon dairy income for inclusion. Eight farms were organically managed while nineteen farms were conventionally managed. Ten interviewees were female and twenty-four were male. Seven interviews interviewed two interviewees, all of which were with one male and one female interviewee in the same age group. One interview involved three interviewees, spanning two age groups.

We employed a combination of snowball sampling [3], online database references, and co-author recommendations to form our sampling pool. Ninety-five farms were contacted with a 28% positive response rate. All interviewees were given a $10 honorarium. Farm size and management practice were the primary farm characteristics used to ensure thorough inclusion of perceptions. Farmer gender, age, and farm generation were recorded but not used to select farms.

Interviews were conducted across management practice, farm size, and farmer age distributions. Twenty-seven total interviews were completed. All interviews were conducted in-person, recorded using a cell phone, and professionally transcribed by the Cornell Institute for Survey and Economic Research. Some interviews were interrupted by third parties with unrelated content. These interruptions were omitted from analysis and largely not transcribed. Upon transcription, interviews were preliminarily coded into the themes by the first author, these themes appear in the headers of each column in the dataset. Within each of these categories, subthemes emerged and were documented by Georgakakos et al [1]. Coding and analysis were preformed using ATLAS.ti 8.4.5 for Mac following the thematic coding methods outlined by Braun & Clarke [4]. The ATLAS.ti software allows filtering of dataset by each theme, which was used to generate the tabulated dataset. The complete codebook code book used for this analysis is reported in supplementary Table 2.

The institutional review board at Cornell University reviewed all study materials and exempt this study from full review. All interview responses have been deidentified, and all potentially identifying information that appeared in the text has been removed.

## Ethics Statement

These data collection and analyses were reviewed by the Institutional Review Board at Cornell University and exempted from full review. All farmers gave informed consent to participate in the interviews and agreed to have interviews recorded, transcribed, and reprinted.

## CRedit Author Statement

**Christine B.Georgakakos:** Conceptualization, Data curation, Formal analysis, Funding acquisition, Methodology, Project administration, Software, Visualization, Writing - original draft; **Betsy Hicks:** Methodology, Project administration, Supervision, Validation, Writing - review & editing; **M. Todd Walter:** Project administration, Supervision, Writing - review & editing.

## Declaration of Competing Interest

The authors declare that they have no known competing financial interests or personal relationships which have or could be perceived to have influenced the work reported in this article.

## Supplementary materials

Image, application 1

## References

[1] Georgakakos C.B., Hicks B.J., Walter M.T. (2000). Farmer perceptions of dairy farm antibiotic use and transport pathways as determinants of contaminant loads to the environment. J. Environ. Manag..

[2] Wemette M., Safi A.G., Beauvais W., Ceres K., Shapiro M., Moroni P., Francis L., Welcome F.L., Ivanek R. (2020). New York State dairy farmers’ perceptions of antibiotic use and resistance: a qualitative interview study. Plos one.

[3] Biernacki P., Waldorf D. (1981). Snowball sampling: Problems and techniques of chain referral sampling. Sociol. Methods Res..

[4] Braun V., Clarke V. (2006). Using thematic analysis in psychology. Qual. Res. Psychol..

