# Supplementary material for: Dairy farmer perceptions of antibiotic transport and usage in animal agriculture dataset
Source: Data Brief. 2021 Jan 22;35:106785. doi: 10.1016/j.dib.2021.106785 (PMC7851767; doi:10.1016/j.dib.2021.106785)
Supplement: Supplementary file 1 [file mmc1.pdf]

Supplementary material

Table S.1. Interview guide.

Interview phase	Questions
Warm up	1) How did you get started farming?
	a. What is the biggest change you've experienced in the past several years in regard to operating your farm?
	b. Farm size: How many <u>*lactating cows*</u> do you have? Total animals? Acreage owned and rented?
	c. How many years have you been working with cattle?
	d. Do you plan on continuing to work with cattle for the foreseeable future?
	e. What step(s) in the dairy production line do you fill?
	f. Do you participate in any dairy or agricultural groups or committees?
	2) Do you have non-dairy centered income? Sell any forage or grains?
	3) Can you describe your method of milk production? *(i.e. conventional, organic, grass-fed, A2, etc.)*
Research Questions	4) How do you manage manure on your farm?
	a. Why do you manage manure the way you do?
	b. Would you like to change your system? If so, what barriers do you face?
	5) Do you use antibiotics to treat your animals?
	a. How do you decide on whether to treat a cow with antibiotics?
	b. How do you decide which specific antibiotic to use for treatment?
	c. Where do you get information to make those decisions?
	d. Are there any sources you avoid for antibiotic information?
	e. How has your usage of antibiotics changed over the last 10-15 years?
	6) Can you list alternative (to antibiotics) medical treatments you use?
	a. How do you decide on whether to treat a cow with alternative medicines?
	b. Where do you get information to make those decisions?
	c. Are there any sources you avoid for medicinal information?
	7) How do you think of antibiotics moving physically around **your/a** farm?
	a. Entry points?
	b. Passage through animal?
	c. Movement with manure?
	d. Do you consider manure management strategies to reduce antibiotic transport/persistence?
	e. What barriers would you encounter changing manure management strategies?
	8) Do you find some categories or life stages of animals require more treatment than others? What are they?
	9) What influences your decisions to **use/not use** antibiotics on your farm in general?
	a. Have consumer opinions and preferences affected you?
	b. Is antibiotic use/resistance something you talk about with others you know in the business?
	c. Are you concerned about anything related to antibiotic resistance in your farm?

	d. Animal recovery?<br>e. Economic reasons?
Additional Info	10) Is there anything we haven't discussed that you would like to include?<br>11) Do you know anyone else who might be interested in participating in this study?

Table S.2. Code book used in generating themes, associated definitions of codes, search words used to assign codes after initial coding, and number of quotations that were tagged with each code. Bolded codes were further subdivided into codes listed within their subsections. Instances where no search words accompany codes indicate codes that were assigned without using search assistant in Atlas.ti.

Code	Definition	Search words	Number of quotations
**Antibiotic usage**		usage	74
Antibiotic usage by others	Perception of antibiotic usage by groups deemed to be the ‘other’		13
Keep cows alive	Usage of antibiotics as a last case scenario, to keep an animal alive	Save her, alive	3
Minimize antibiotic usage	Farmer perspective that they minimize usage of antibiotics	Minimize;	7
Organic cow = sick cow	Perspective that organic cows tend to be sicker than conventional	Organic cow	3
**Antibiotic Transport**	Movement of antibiotic compounds around the farm, after an animal is treated	Transport	46
Antibiotics in manure	Transport of antibiotics broadly with manure	Manure	27
Antibiotics in manure – no	Farmer does not think about transport of antibiotic with manure	Manure	15
Antibiotics in manure – yes	Farmer thinks about transport of antibiotics with manure	Manure	7
Antibiotics in meat	Perspective on antibiotic presence in meat	Meat, beef	11
Dead animal disposal	Discussion of what is done with carcasses on farm	render, dead, mortality	
Market milk	Milk shipped for sale	Milk	25
Waste milk	Milk not sold but sometimes fed to calves	Waste milk, fed to calves	16
**Alternatives to antibiotics**	Therapies used or actions taken that can result in reduced antibiotic usage.	Alternative, preventative	62
Facility conditions	Reference to facility conditions of the cows	Facility, ventilation, building, cow comfort	19
Nutrition	Importance of feed in maintaining healthy cows	Nutrition, diet, feed*	
Calf Health & Nutrition	Considerations specific to calves for nutrition and supplements	calf, calves	33
Preventative action for sickness	Actions taken to reduce disease		30
Stress reductions	Stress reductions, generally around not pushing cows for production	stress	5
Vaccines	Usage of vaccines as a disease prevention	Vaccin*	24
Culling cows for medical reasons	Culling cows as related to reducing disease presence and antibiotic usage	Cull*, call+, cold+, sell for beef	17
Herbals	Supplements used as a therapy in place of an antibiotic	Herb*, garlic, mint	34
Minerals	Mineral supplements given to fresh cows	Mineral, calcium	12

**Manure Management**	Manure management system on the farm	Manure	46
Money is a barrier to changing the system	Funding as a barrier to implementing another management system	Manure, money	12
Nutrient management considerations	Nutrient management for pollution or usage on crops	Manure, nutrient	16
Regulations require this system	Regulations requiring farms to use specific manure management	Manure, regulation, CAFO	7
This is the system we have	Current system is used primarily out of convenience	Manure	11
Desired changes to manure management system	Any desired change to their manure management system	Manure	39
Manure management considerations around antibiotics	Manure management decisions drive by reducing antibiotic residue spread	Manure	10
**Antimicrobial Resistance**	Perspectives of antimicrobial resistance	Resistance	44
Concerned about AMR on this farm	Farmers expressed concern about AMR on their farm	Resistance	14
Concerned about AMR, but not on this farm	Farmers expressed concern in general about AMR, but do not think it is a problem on their farm	Resistance	7
Concerned about human resistance	Farmers expressed concern about human antimicrobial resistance, related or not to usage of antibiotics in agriculture	Human, anthropogenic, resistance	8
Not concerned about AMR	Farmer did not express concern about AMR specifically or in general	Resistance	7
**Alternative Information**	Sources trusted or relied on for alternative information	Info*	46
Books, positive		Book, info	4
Extension, positive	,	extension, info	1
Internet, positive		internet, info	5
Lack of knowledge	Identification of a lack of information on alternatives as a suggestion to why they may not be used		1
Magazines, positive		magazine, newsletter, publication, info	7
Milk shippers, positive		organic valley, co-op, coop, info	4
People, positive		personal experience, info	17
Product companies, positive		compan*, info	5
Sources avoided	Information sources actively avoided or not trusted	avoid, info	13
Trainings, positive		training, meeting	7
Vets, positive		vet*, info	3

**Antibiotic information**	Sources trusted or relied on for antibiotic information	Info*	55
Books, positive		Book, info	1
Companies, positive		compan*, info	6
Extension, positive		extension, info	2
Internet, Negative		internet, info	3
Internet, positive		internet, info	4
Lack of knowledge			1
Magazines, negative		magazine, newsletter, publication, info	3
Magazines, positive		magazine, newsletter, publication, info	3
Milk shippers, positive		organic valley, co-op, coop, info	1
Open to all	Identification that the farmer is open to all sources, once investigated		1
People, positive		personal experience, info	14
Pharmaceutical companies, negative		drug, compan*, info	5
Product Label, positive		label, bottle, info	4
Sources avoid		avoid, info	17
Trainings, positive		train*, meeting	2
Vet, positive		vet*, info	29

* Search word roots that were used to be inclusive of more than one word

$^{+}$ Term that was incorrectly transcribed in place of 'cull'.
